# Role of the blue light receptor gene *Icwc-1* in mycelium growth and fruiting body formation of *Isaria cicadae*

**DOI:** 10.3389/fmicb.2022.1038034

**Published:** 2023-01-10

**Authors:** Linhao Song, Neeraj Shrivastava, Yunpeng Gai, Dong Li, Weiming Cai, Yingyue Shen, Fu-Cheng Lin, Jingyu Liu, Hongkai Wang

**Affiliations:** ^1^State Key Laboratory of Rice Biology, Institute of Biotechnology, Zhejiang University, Hangzhou, China; ^2^Shanxi Key Laboratory of Edible Fungi for Loess Plateau, College of Food Science and Engineering, Shanxi Agricultural University, Taigu, Shanxi, China; ^3^Amity Institute of Microbial Technology, Amity University, Noida, Uttar Pradesh, India; ^4^School of Grassland Science, Beijing Forestry University, Beijing, China; ^5^Institute of Horticulture, Zhejiang Academy of Agricultural Sciences, Hangzhou, China; ^6^State Key Laboratory for Managing Biotic and Chemical Treats to the Quality and Safety of Agro-Products, Institute of Plant Protection and Microbiology, Zhejiang Academy of Agricultural Sciences, Hangzhou, China

**Keywords:** *Isaria cicadae*, blue light receptor, mycelium growth, fruiting body formation, developmental regulation

## Abstract

The *Isaria cicadae*, is well known highly prized medicinal mushroom with great demand in food and pharmaceutical industry. Due to its economic value and therapeutic uses, natural sources of wild *I. cicadae* are over-exploited and reducing continuously. Therefore, commercial cultivation in controlled environment is an utmost requirement to fulfill the consumer’s demand. Due to the lack of knowledge on fruiting body (synnemata) development and regulation, commercial cultivation is currently in a difficult situation. In the growth cycle of macrofungi, such as mushrooms, light is the main factor affecting growth and development, but so far, specific effects of light on the growth and development of *I. cicadae* is unknown. In this study, we identified a blue light receptor white-collar-1 (*Icwc-1*) gene homologue with well-defined functions in morphological development in *I. cicadae* based on gene knockout technology and transcriptomic analysis. It was found that the *Icwc-1* gene significantly affected hyphal growth and fruiting body development. This study confirms that *Icwc-1* acts as an upstream regulatory gene that regulates genes associated with fruiting body formation, pigment-forming genes, and related genes for enzyme synthesis. Transcriptome data analysis also found that *Icwc-1* affects many important metabolic pathways of *I. cicadae*, i.e., amino acid metabolism and fatty acid metabolism. The above findings will not only provide a comprehensive understanding about the molecular mechanism of light regulation in *I. cicadae*, but also provide new insights for future breeding program and improving this functional food production.

## 1. Introduction

Fungi are the second largest species on the earth after insects, with abundant resources. Entomopathogenic fungi is an important branch among them, which has special value in production for human health. *Isaria cicadae*, is an edible and potent medicinal entomopathogenic fungus with lots of immunogenic properties. The fruiting bodies (synnemata) of *I. cicadae* are collected due to its multiple pharmacological attributes and unique flavor. These fruiting bodies of *I. cicadae* have abundant important constituents such as cordycepic acids (Sun et al., 2017), cordycepin (Olatunji et al., 2016a; Zhang et al., 2019), polysaccharides (Jike et al., 2016; Wang et al., 2019), adenosine (Latini and Pedata, 2010; Olatunji et al., 2016b), ergosterol peroxide (Kuo et al., 2003), myriocin (ISP-1; Yu et al., 2009; Fujita et al., 2010), etc. These ingredients show important pharmaceutical properties such as antitumor (Kuo et al., 2003), anti-influenza (Lu et al., 2015), and anti-inflammatory responses (Smiderle et al., 2014; Jiao et al., 2021). Over the past few decades, *I. cicadae* has become one of the most interesting research topics in the field of natural traditional medicines worldwide. Studies have shown that some ingredients of *I. cicadae* have a significant clinical effect in treating nephropathy also (Li et al., 2019; Huang et al., 2020).

*I. cicadae* has many similarities with *Ophiocordyceps sinensis* in various components and functions, and can be used as a substitute for the expensive traditional Chinese medicine (Zeng et al., 2014). In addition, recently, *I. cicadae* was listed as a novel food by the Ministry of Health of the People’s Republic of China^1^. However, due to the rapid reduction of natural resources and the long life cycle, there is a serious shortage of wild *I. cicadae* resources and the market needs are not meet (Liu, 2008). Moreover, the lack of knowledge on the developmental regulations and conditional development of *I. cicadae* fruiting bodies, have negatively impacted large-scale commercial production of *I. cicadae*. As a result of these constraints, there is an urgent need to focus on the developmental regulation of the fungus to meet the demand for artificial culture. The mechanism of fruiting bodies formation has great significance in the cultivation and breeding of artificially grown edible mushrooms. The mechanism of morphogenesis of fungi, especially large edible basidiomycetes, has always been one of the hot topics in mycological researches (Wu et al., 2019). To understand the mechanism behind this phenomenon, researchers worked on model fungi *Schizophyllum commune* and *Coprinus Coprinopsis cinerea* to gain further mechanistic insight (Terashima et al., 2005; Ohm et al., 2013). Yet, there is still a lack of clarity about the mechanism of *I. cicadae* fruiting body formation and development to scale up the production to commercial level.

Light is one of the most important environmental factors in the life cycle of fungi and play an important role in growth and metabolism. A majority of the fungi respond to light, eliciting changes in several physiological characteristics including pathogenesis, development and secondary metabolism. Fungi respond to light by photoreceptor proteins where the light-absorbing component undergoes a photochemical and structural changes. In *Neurospora crassa*, the light-induced changes are transduced from photoreceptor proteins to a signaling cascade that modulates downstream pathways by White Collar-1 (WC-1) protein. *N. crassa* has been shown to respond to blue light, and this response is mediated by the WC-1 and WC-2 proteins acting in a complex called the White-Collar-Complex (WCC). WCC functions as light-activated transcription factor (Dasgupta et al., 2016). In recent years, many researchers have focused on exploring signal transduction pathways of fungal photosensitive mechanisms. In filamentous fungi, the mechanism of action of the *wc-1* gene has been investigated more clearly (Estrada et al., 2008). Knockout mutants of the *wc-1* and *wc-2* genes in *N. crassa* are incapable of light response, including defect in the synthesis of carotenoids in mycelia, circadian clock dysregulation, and loss of phototropism in the conidia beak (Linden et al., 1997; Liu et al., 2003). Subsequent experiments have shown that the *wc-1* and its homologs are also found in macrofungi, including *Coprinus cinereus* (Terashima et al., 2005), *Schizophyllum commune* (Ohm et al., 2013), *Cordyceps militaris* (Yang and Dong, 2014), and *C. sinensis* (Yang et al., 2013). Research results showed that in some edible fungi, the functions of WC-1 homologs are related to the fungal growth and development. Hence, it is implied that *wc-1* gene might have an important influence on fungal growth and development (Ohm et al., 2013).

In this study, by following genetic approaches and analyzing transcriptomic data in *I. cicadae*, we confirmed that the *Icwc-1*, the photoreceptor gene, plays a positive role in regulating the growth and development in *I. cicadae.* This study will also provide important information for *I. cicadae* breeding, production process improvement and development of related functional foods.

## 2. Materials and methods

### 2.1. Strains and growth conditions

The wild strain of *I. cicadae* (WT) was provided by College of Agriculture and Biotechnology, Zhejiang University, PR China. The *Icwc-1* knockout mutant (∆*Icwc-1*) strain and the complementary strain (∆*Icwc-1-*C) were developed in this study. Plasmid pCAMBIA1300 (Liu et al., 2015), used for knockout vector and PKD5-GFP (Qu et al., 2022), used for complementary vector, were procured from Zhejiang University. The competent cells of *Escherichia coli* DH5α and *Agrobacterium tumefaciens* AGL-1 were purchased from Qingke (Hangzhou) Biotechnology Co., Ltd., PR China.

All the *I. cicadae* strains were routinely maintained on Potato Dextrose Agar (20% potato, 2% D-glucose, 1.5% agar) at 25°C. *E. coli* and *A. tumefaciens* were grown in Luria-Bertani (LB) broth (1% NaCl, 0.5% yeast extract, and 1% tryptone) or LB agar. Auto-induction medium and co-cultivation medium (AIM) were used for *A. tumefaciens*-mediated transformation (ATMT) of *I. cicadae* (Khang et al., 2006).

### 2.2. Bioinformatics analysis

The nucleotide sequence of *Icwc-1* gene was obtained from the genome of *I. cicadae* strain WT. Using BLAST from NCBI, the nucleotide sequence similarity was analyzed^2^. Protein motifs were identified using the Conserved Domain Database from NCBI. The amino acid sequence of IcWC-1 protein in *I. cicadae* were predicted from the genome of the wild-type (WT) strain, and the other species WC-1 protein sequences were obtained from NCBI database. The Neighbor Joining method were used to generate the homologous evolutionary tree of WC-1 protein sequence on MEGA7 version software (Kumar et al., 2016).

### 2.3. Disruption of *Icwc-1* gene in *Isaria cicadae*

Genomic DNA was prepared using the cetyltrimethylammonium bromide (CTAB) method (Doyle and Doyle, 1987). Primers *Icwc-1*-F/R was used to amplify the full-length *Icwc-1* gene. A strategy of homologous recombination was employed to delete the *Icwc-1* in *I. cicadae*. The 1,185 bp and 1,286 bp DNA fragments upstream and downstream of *Icwc-1* gene were amplified from genomic DNA with primers *Icwc-1*-UP- F/R and *Icwc-1*-DOWN-F/R, respectively. The hygromycin phosphotransferase gene *hph* fragment was cloned from plasmid pBHt2. These three fragments were connected by Fusion Enzyme Kit (Vazyme, China) and inserted into the vector pCAMBIA1300 digested with *Xho*l and *Hind*III, to generate pCAMBIA1300-*Icwc-1* knockout vector.

The constructs were introduced into *I. cicadae* by ATMT using the method reported by (Khang et al., 2006) with slight modifications. Conidia for transformation were harvested and suspended into the sterile 0.05% Tween 80 and adjusted to a concentration of 10^5^ spores/mL. Further, 100 μl of *I. cicadae* conidial suspensions and 100 μl of *A. tumefaciens* were mixed together and spread on the AIM agar plate and co-incubated at 23°C for 2 days. The co-culture of *A. tumefaciens* and *I. cicadae* was covered with PDA agar supplemented with 300 μg/ml cefotaxime and 350 μg/ml hygromycin (hygB) and incubated at 23°C for 3–6 days. Primers *Icwc-1*-CK-F/R were used to identify the transformants by PCR. All primers used in this study are listed in the Supplementary Material Table S1.

### 2.4. Complementation of the *Icwc-1* disruption mutant

To investigate the function of the *Icwc-1* gene, the complementary experiment of the *Icwc-1* gene was carried out. Primers *Icwc-1*-HB-F/R was used to amplify the *Icwc-1* gene (containing PKD5-GFP vector linker) in the genome of wild-type strain of *I. cicadae*. The PKD5-GFP vector digested with *Xba*l and *Sal*I, and the full-length *Icwc-1* gene, the 2,940 bp fragment, were inserted into the corresponding sites of GFP to generate PKD5-GFP-*Icwc*-1. For complementation, PKD5-GFP-*Icwc*-1 was introduced into the ∆*Icwc-1* strain by the ATMT method. Transformants were selected on DCM plate supplemented with 200 μg/ml of SUR at 25°C. The complemented strain ∆*Icwc-1-*C was confirmed by PCR amplification using primer paire Icwc-CoF and Icwc-CoR.

### 2.5. Mutant transformants validation by southern blotting and q-PCR

For Southern blot analysis of genomic DNA, 50 μg of DNA extracted from each three mutant strains and wild-type strain were digested with *Xho*I restriction enzyme and separated on 0.7% agarose gel. The mutant *hph* fragment was amplified from pCAMBIA1300 plasmid using primers Southern-F and Southern-R as probes. The probe labeling, hybridization and signal detection were performed by employing a DIG DNA Labelling and Detection Kit (Cat. No. 11745832910, Roche, Germany).

In addition, single copy of knock-out mutants were also confirmed by q-PCR method according to the assay descripted by Lu et al., 2014 (Lu et al., 2014). Briefly, when tubulin as the reference gene, single copy of the target gene was determined as ∆∆CT = 0.9 ~ 1.3, where ∆∆CT = (CT_HPH_–CT_tubulin-m_) − (CT_gene_–CT_tubulin-w_), CT_HPH_ is the CT value of HPH in mutant, CTt_ubulin-m_ is the CT value of tubulin in mutant, CT_gene_ is the CT value of target gene in wild-type strain, CT_tubulin-w_ is the CT value of tubulin in wild-type strain.

### 2.6. Measurement of hyphal growth rate, spore production and biomass

Three *I. cicadae* strains, WT, ∆*Icwc-1*, ∆*Icwc-1-*C, were maintained at 25°C on PDA medium. The spores were gently washed with ultra-pure water and diluted to 1 × 10^6^/mL. The 10 μl spore suspension was inoculated at the center of PDA containing petri-plates. These culture plates were incubated at 25°C and after 4 days of growth, the colony edge was marked and measured every 24 h till next 7 days under the light and dark (12 h:12 h) culture conditions. During these intervals of time, the spores were washed and counted. This experiment was repeated three times independently. 5 μl spore suspension was added to PDA medium containing cellophane, incubated for 7 days under white light conditions, hyphae collected, baked to constant weight in a 65°C oven, and data was recorded.

### 2.7. RNA preparation and RT-PCR analysis

The 10 μl spore suspension was inoculated in PDB liquid medium, cultured at 25°C with 140 rpm under the light and dark (12 h:12 h) culture conditions and samples were collected after 7 days. Mycelium (0.2 g) was ground to powder with liquid nitrogen, and RNA was extracted using RNAiso Plus (TaKaRa, Japan) according to manufacturer’s instructions. Reverse transcription of total RNA was carried out using PrimeScripTM RT regent Kit with gDNA Eraser (Takara, Japan). The qRT-PCR was then performed using TB Green^®^ Premix EX TaqTM (Tli RNaseH Plus; TaKaRa, Japan) to analyze the expression level of development-related genes. All primers used in this study are listed in the Supplementary Material Table S1.

### 2.8. Cultivation methods for fruiting bodies

For culturing *I. cicadae*, wheat medium as described by Guo et al. (2016), was used. The 10 μl of the spore suspension of wild-type strain WT, mutant strain ∆*Icwc-1*, and the complemented strain ∆*Icwc-1*-C, were inoculated on wheat medium separately and cultured in dark at 25°C until mycelium-covered the medium. The mycelial colonized substrate was transferred to a culture box with alternating light and dark (12 h:12 h) for 1 month. The growth status of the strains was observed and photographed.

### 2.9. RNA seq analysis

For RNA-Seq analysis, the Illumina NovaSeq platform was used for paired-end sequencing of wild type and null mutant ∆*Icwc-1*. The RNA-Seq raw reads obtained by sequencing on the Illumina sequencing platform were processed to remove adaptors and low-quality bases using Trimmomatic v0.39 (Bolger et al., 2014) with default parameters (ILLUMINACLIP:adapters:2:30:10 SLIDINGWINDOW:4:20 MINLEN:50). In order to identify the genes with low expression or only partial fragments in individual RNA-Seq samples, the clean reads of all samples were assembled to a reference sequence using Trinity v2.8.5 (Grabherr et al., 2011). Further, Corset v1.09 (Davidson and Alicia, 2014) was used to aggregate transcripts into many clusters according to the Shared Reads between transcripts, and then combined the transcript expression levels between different samples and the H-Cluster algorithm to classify the expression differences between samples. The longest transcript in each cluster is selected as the representative sequence in the cluster, which is defined as the unigene sequence. To obtain comprehensive gene function information, the transcripts were functionally annotated to obtain the functional information of the gene from different databases, including: Nr (NCBI non-redundant protein sequences), Nt (NCBI nucleotide sequences), KOG (Clusters of Orthologous Groups of proteins), Swiss-prot (A manually annotated and reviewed protein sequence database), Uniprot (Universal Protein), KEGG (Kyoto Encyclopedia of Genes and Genomes), GO (Gene Ontology) using emapper v2.0.0 (Cantalapiedra et al., 2021). The cleaned reads were subsequently mapped to the reference sequences assembled by Trinity using bowtie2 (Langmead and Salzberg, 2011). To quantify the gene expression levels, the number of clean reads mapped to a gene is called read count of each sample using RSEM v1.2.28 (Cantalapiedra et al., 2021). The R package DESeq2 was used to identify differentially expressed genes (DEGs) under the threshold of FDR ≤ 0.05 and absolute value of log2FC ≥1. The GO enrichment was performed using the R package cluster Profiler (Yu et al., 2012).

## 3. Results

### 3.1. The structural characters of *Icwc-1* gene and phylogenetic analysis of the WC-1 proteins in related fungal species

**Figure 1:**
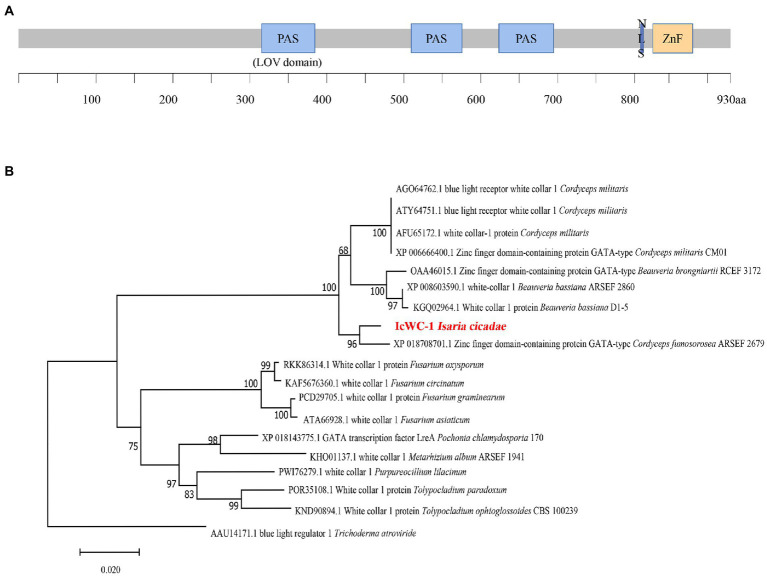
The domain organization of IcWC-1 protein and the Phylogenetic tree of IcWC-1. **(A)** The regions corresponding to LOV, PAS domains, the nuclear localization signal (NLS) and ZnF are shown. **(B)** Phylogenetic tree of WC-1 homologous proteins from fungi. The amino acid sequences of WC-1 homologous proteins from different species were downloaded from the NCBI database for phylogenetic analyses (protein sequences in Supplementary Material). The topology of this tree was generated using neighbour-joining (NJ) method of MEGA7 with 1,000 bootstraps replicates. These numbers represent the percentage of replication trees (1,000 replications) with related taxa clustered together in the boot test.

The nucleotide sequence of *Icwc-1* gene with a length of 2,940 bp was identified from the genome of *I. cicadae*. The *Icwc-1* gene contains an open reading frame of 2,878 bp, interrupted by one intron of 62 bp (Supplementary material), and encodes a protein with 959 amino acid residues (see Supplementary material for Amino acid sequence of WC-1 protein of *I. cicadae*). The sequence information of *Icwc-1* gene was stored in Genbank with accession number OP675621. The Domain prediction with EXPASY, showed that the IcWC-1 protein has three PAS (Per-Arnt-Sim) structural domains. The first domain is a LOV structural domain (light, oxygen, voltage; Figure 1A), second domain is a ZnF (zinc-finger DNA-binding motif) and third one is a GATA structural domain. In fungi, the LOV domain conserves and can receive light signals as a receptor, while the ZnF and GATA type domain is a transcription factor that specifically recognizes and regulates the GATA sequence of promoters of downstream gene regulatory regions, suggesting that in *I. cicadae*, *Icwc-1* may be a receiver of light signal, which in turn regulates the expression of downstream genes.

In addition, we performed phylogenetic analysis of the white collar 1 (WC-1) protein of 19 fungi, which showed that the WC-1 proteins of these species are indeed evolutionarily similar, and the homology between *I. cicadae* and *Cordyceps fumosorosea* ARSEF 2679 was higher (Figure 1B). The results showed that *I. cicadae* was more closely related to *C. fumosorosea* ARSEF 2679 (ISF_00644) in evolutionary relationship.

### 3.2. Generation of mutants for *Icwc-1* knockout and complement

**Figure 2:**
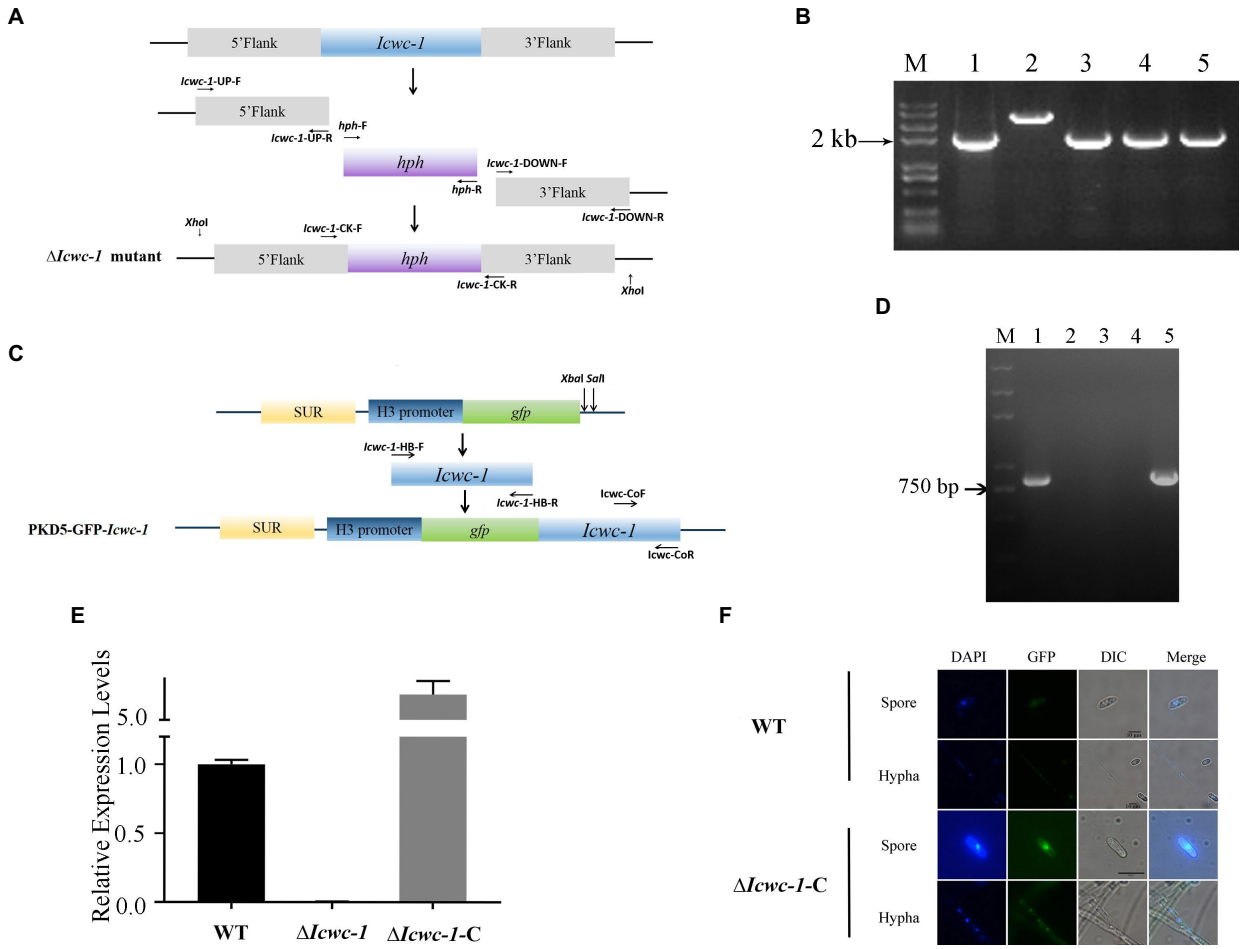
Generation and identification *Icwc-1* mutants of *I. cicadae*. **(A)** Schematic diagram of knockout ∆*Icwc-1 via* homologous recombination. **(B)** PCR validation of knockout strains. M: Maker, 1: knockout vector PCAMBIA1300-*Icwc-1*, 2: WT strain, 3–5: mutants. 3: ∆*Icwc-1-*2, 4: mutant ∆*Icwc-1-*8, 5: mutant ∆*Icwc-1-*40. **(C)** Schematic diagram of the generation of the complemented vector. H3 promoter in pKD5-GFP derived from *Pyricularia oryzae*. **(D)** PCR verification of complementary strains. M: Maker, 1: wild type strain WT, 2: mutant strain ∆*Icwc-1-*2, 3: mutant strain ∆*Icwc-1-*8, 4: mutant strain ∆*Icwc-1-*40, 5: the complementary strain ∆*Icwc-1-*C. **(E)** Expression of *Icwc-1* gene in wild-type strain, mutant strain, and the complementary strain. **(F)** Location of *Icwc-1* gene expression analysis. GFP observation under fluorescence microscope. The hyphae and spores of the wild-type strain and the ∆*Icwc-1*-C strain were stained with DAPI and observed under a fluorescence microscope. Bar = 10 μm.

To investigate the biological functions of *Icwc-1* in *I. cicadae*, the *Icwc-1* knockout vector pCAMBIA1300-*Icwc-1* and the *Icwc-1* complement vector pkd5- *Icwc-1-gfp* were constructed (Figures 2A,C). The pCAMBIA1300-*Icwc-1* was transformed into the *I. cicadae* wild-type strain through ATMT method. Putative strains of ∆*Icwc-1* were confirmed by PCR analysis. DNA fragments of 3,969 bp and 2,383 bp were uniquely amplified with the primer sets *Icwc-1*-CK-F and *Icwc-1*-CK-R from the wild-type and ∆*Icwc-1* strains, respectively (Figure 2B). Southern blot analysis (Supplementary Figure S1) showed the disruption of the *Icwc-1* locus and the homokaryotic nature of the mutant strains. Single copy knock-out mutant was also verified by Q-PCR where the ∆∆CT value was 0.9. In a word, PCR identification, southern blotting, and q-PCR results all showed that we had successfully obtained a homokaryotic mutant of *Icwc-1*. The mutant ∆*Icwc-1-*40 was used to analyze the phenotypes in the development process of fungi (Figure 2D).

To further confirm the biological role of *Icwc-1* gene, we generated the complemented strain ∆*Icwc-1*-C, in which the full-length *Icwc-1* gene were introduced into the ∆*Icwc-1* mutants, and verified by PCR and RT-PCR. The 0.8 kb of *Icwc-1* fragments were amplified in ∆*Icwc-1*-C strain and wild strain (Figure 2D), indicating that *Icwc-1* was successfully replenished into the *ΔIcwc-1* mutant. The RT-PCR analysis showed that *Icwc-1* was not expressed in mutant ∆*Icwc-1*, while the expression of the *wc-1* gene in the complementary strain ∆*Icwc-1*-C was restored (Figure 2E). All in all, ∆*Icwc-1*-C was replenished into the mutant strain, and also showed the correctness of the mutant strain. GFP observation confirmed that Icwc-1 protein was located in nucleus (Figure 2F).

### 3.3. Role of *Icwc-1* gene on the growth characters and colony morphology

**Figure 3:**
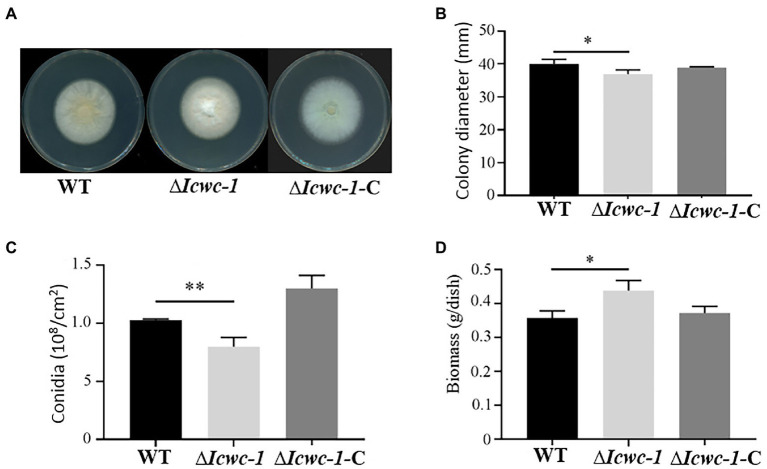
Effect of *Icwc-1* gene on growth characters in *I. cicadae.* **(A)** Colony characters of different strains (wild-type, ∆*Icwc-1* and ∆*Icwc-1*-C) of *I. cicadae* were photographed and recorded after 10 days of alternating light and dark culture on PDA medium; **(B)** 5 μl of the spore suspension is inoculated on PDA medium, cultured under white light conditions for 10 days, and the colony diameter was recorded; **(C)** The strains were cultured on a PDA plate for 10 days at 25°C, spores were washed with water, diluted to a certain concentration and counted under a microscope; **(D)** Effects of *Icwc-1* gene deletion on mycelial biomass of *I. cicadae.* The symbol “*” indicates a significant difference (*p* < 0.05) compared with wild-type strain. The symbol ‘**’ indicates significant difference (*p* < 0.01) compared with wild-type strain.

To investigate the growth characters and pattern of fungal growth, the colony diameters of the knockout strain ∆*Icwc-1*, the complementary strain ∆*Icwc-1-*C and the wild type were measured at 10 days after inoculation, and all results of triplicate experiments were compared and statistically analyzed (Figure 3). At the 10th day of incubation, significant differences (*p* < 0.05) in colony morphology and diameter of all the three strains were observed. Among them, the colony diameter of ∆*Icwc-1* was smaller and spore production was also reduced compared to WT and complementary strain ∆*Icwc-1-*C. The results indicated that the *Icwc-1* gene of *I. cicadae* has a regulatory effect on both of the growth rate of the fungal mycelium and sporulation (Figure 3B).

In terms of colony morphology, we found that the mycelium of the wild-type strain WT was sparse, thin, yellowish, and mainly creeping on the medium. However, the mycelium of the ∆*Icwc-1* strain was thicker, mountainous, and mainly aerial on the medium, with white mycelium (Figure 3A), indicating that the *Icwc-1* gene had a greater effect on the aerial hyphal growth and colony morphology.

Sporulation is an important means of reproduction in *I. cicadae*. During the study, it was found that when the *Icwc-1* gene was knocked out, the number of spores of the mutant strain ∆*Icwc-1* was significantly reduced as compared to the wild-type strain WT and the complementary strain ∆*Icwc-1-*C, while the number of spores of the wild-type strain WT and the complementary strain ∆*Icwc-1-*C did not differ significantly (Figure 3C), indicating the influence of *Icwc-1* gene on sporulation of the fungus. The reduction of spores in null mutant may have resulted due to decrease of conidiophore compared to the WT strain. Due to deletion of the *Icwc-1* gene, indicating that the *Icwc-1* gene of *I. cicadae* positively regulates the conidial spore formation pathway.

The biomass is an essential component for the large-scale production of *I. cicadae*. The results of biomass determination experiments showed that when the *Icwc-1* gene was knocked out, the biomass of the strain increased significantly, possibly due to the increase in aerial hyphae of the mutant strain ∆*Icwc-1*, indicating that the *Icwc-1* gene may have a significant inhibitory effect on the production of hyphae biomass in *I. cicadae* (Figure 3D).

### 3.4. Impact of *Icwc-1* on carotenoid synthesis genes

**Figure 4:**
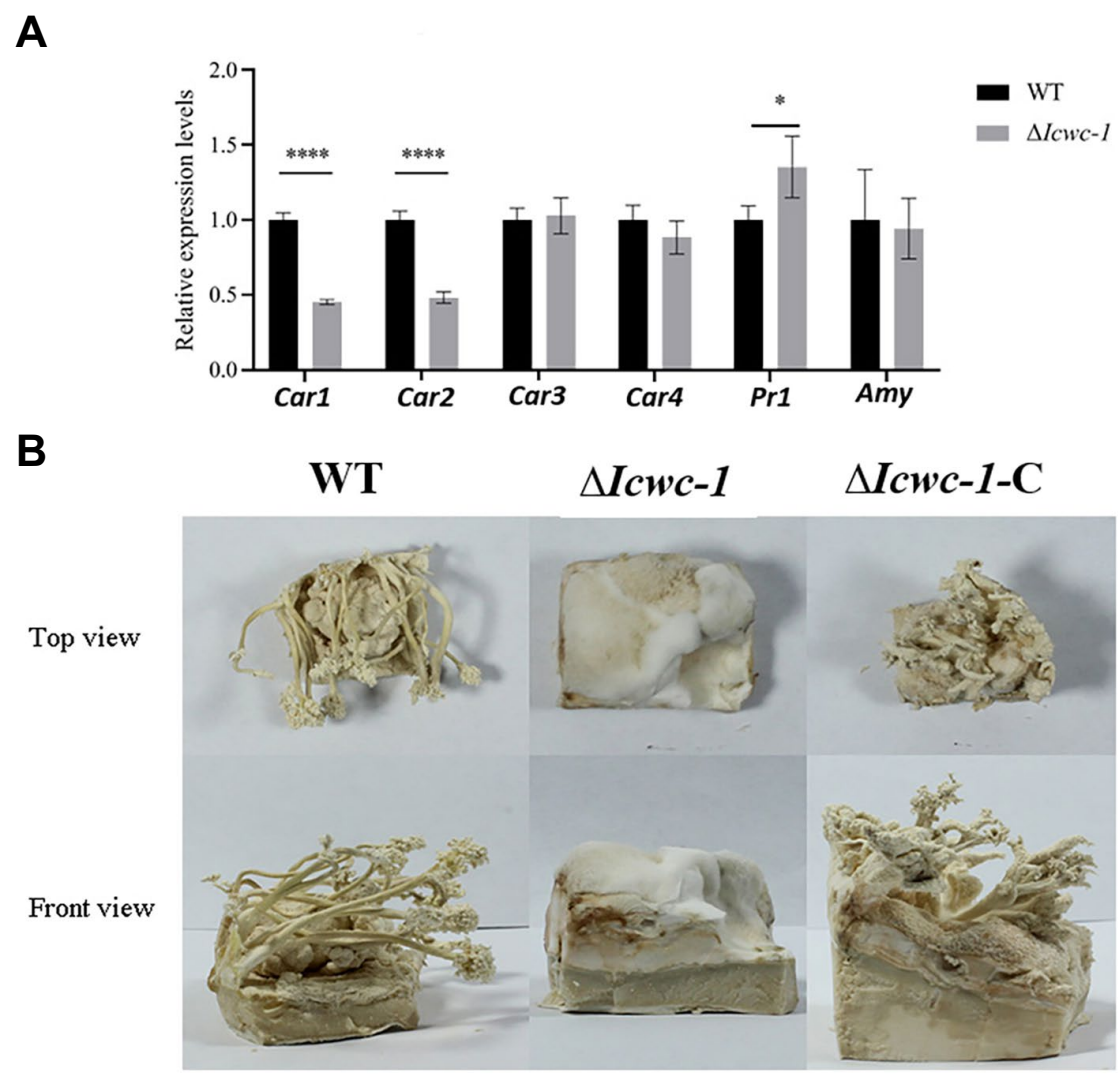
Effect of *Icwc-1* gene deletion on expression of genes related to mycelial metabolism and fruiting body formation of *I. cicadae*. **(A)** The relative expression of genes of wild-type strain and *Icwc-1* mutant strain in *I. cicadae* detected by RT-PCR. **(B)** Fruiting body formation of *I. cicadae* (WT, ∆*Icwc-1* and ∆*Icwc-1*-C). The symbol “*” indicates a significant difference (*p* < 0.05) compared with the original strain, while “****” indicates an extremely significant difference (*p* < 0.01). Gene expression profile regulated by *Icwc-1* gene.

In fungi, carotenoids are an important class of metabolites, and light is an important environmental factor that induces the carotenoid biosynthesis. As the carotenoid synthesis pathway is still unclear in this fungus, we found 4 homologous genes with crucial role of carotenoid biosynthesis by screening genome of *I. cicadae*. Among them, *Car1* is homologous with ylo-1 in *Neurospora crassa* (Estrada et al., 2008), *Car2, Car3 and Car4* are homologous with CCM_03059, CCM_03697, and CCM_06355 in *Cordyceps militaris, respectively,* (Lou et al., 2019), which are the key genes in the biosynthesis of carotenoids pathway. The expression levels of the key genes in the carotenoid synthesis pathway may be reflected in the content of carotenoids *in vivo*. RT-PCR results showed that the expression of *Car1* and *Car2* in the mutant strain ∆*Icwc-1* was down-regulated by about 50% as compared to the wild-type. However, there was no significant change in the expression level was observed in *Car3* and *Car4* (Figure 4A).

### 3.5. Impact of *Icwc-1* on protease and amylase

Protease and amylases are important enzymes in fungal physiology, and play an important role in the invasion of *I. cicadae* into host and parasitic growth. Both enzymes help *I. cicadae* to break down the host proteins and carbohydrates for better nutrient absorption. RT-PCR was used to detect the expression levels of *prl* and *amy* genes in wild-type and mutant strains, and it was found that the expression level of *pr1* gene in mutant strain ∆*Icwc-1* was 1.37 times higher than wild-type strains. There was no significant difference in the expression of the *amy* gene in the mutant strains (Figure 4A). The experimental results show that *Icwc-1* may mainly affect the expression of protease to achieve the purpose of infection during *I. cicadae* infestation in the dark, this result may adapt the habit that this entomopathogenic fungus infects cicada nymphs underground.

### 3.6. Role of *Icwc-1* gene on fruiting body formation

Since *I. cicadae* is full of medicinal properties, so for commercial production of fruiting body of this mushroom, we need to pay attention on the mechanism of fruit body formation and factors responsible for the growth and development. To investigate whether the *Icwc-1* gene influences the formation of fruiting bodies of *I. cicadae*, cultivation experiments were performed on artificial flour medium. The wild type, mutant and complementary strains were inoculated on flour medium and incubated in the dark until the mycelium covers the medium. Later, the strains were placed in the light for pin head formation and development of fruiting body. The experimental results showed that the complementary strain ∆*Icwc-1*-C could form fruiting bodies, like the wild-type strains, but when the *Icwc-1* gene was knocked out, the strain could only form some aerial hyphae on the flour medium and could not differentiate into fruiting bodies (Figure 4B). These results show that the *Icwc-1* gene is a necessary for the growth and development of fruiting bodies in *I. cicadae*.

### 3.7. Transcriptomic analysis of ∆*Icwc-1* mutant

**Figure 5:**
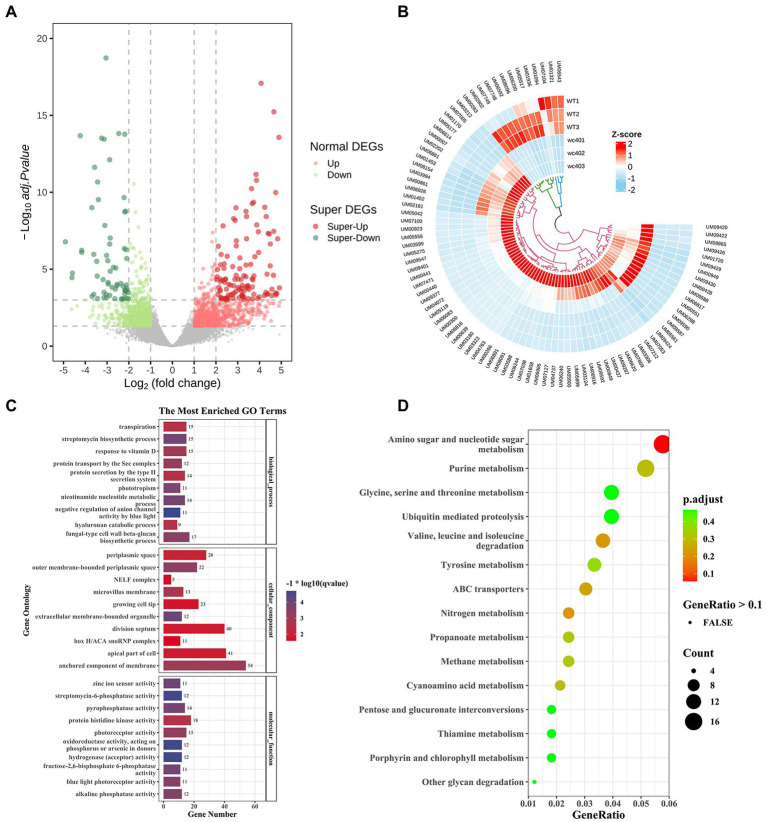
Comparative gene ontology (GO) analysis of the differentially expressed genes (DEGs) between the transcriptome of wild type and *Icwc-1 mutants.* **(A)** The volcano plot is two-dimensional, with the *y*-axis representing the negative log10 of the adjusted value of *p*, and the *x*-axis displays the variation ratio of each gene (log2 FC). The green spots represent significantly down-regulated genes in the mutants, while the red spots indicate up-regulated genes [false discovery rate (FDR) < 0.05]. Gray spots represent the genes that did not show differential expression. **(B)** The heat map shows the gene expression patterns in *Icwc-1* mutants and wild-type (WT) of *I. cicadae*. **(C)** The results are summarized in three main GO categories (Cellular Component, Molecular Function and Biological Process). The *x*-axis indicates different mutants. The *y*-axis indicates the GO term. **(D)** KEGG enrichment analysis of the DEGs between the transcriptome of *I. cicadae* wild-type and *Icwc-1* mutants. The *y*-axis indicates the KEGG pathway. The *x*-axis indicates the gene ratio.

To explore the molecular mechanisms underlying that the white collar-1 (*Icwc-1* gene) regulating the formation of fruit-body in *I. cicadae*, we performed transcriptomic analysis comparing the gene expression of the wild-type strain and ∆*Icwc-1* gene mutants during asexual development. Overall, 2009 genes were differentially expressed in ∆*Icwc-1* compared with the wild-type strain (Supplementary Table), comprising 947 up-regulated genes and 1,062 down-regulated genes (Figure 5A; Supplementary Tables S2–S5). GO enrichment analysis (Supplementary Tables S3–S5) revealed that the inactivation of *Icwc-1* significantly affected the expression of many genes enriched in the molecular function (MF), including “oxidoreductase activity, acting on phosphorus or arsenic in donors (12),” “hydrogenase (acceptor) activity (12),” “streptomycin-6-phosphatase activity (12),” “zinc ion sensor activity (11),” “fructose-2,6-bisphosphate 6-phosphatase activity (11),” “pyrophosphatase activity (14),” “alkaline phosphatase activity (12),” “blue light photoreceptor activity (11),” “photoreceptor activity (15),” “protein histidine kinase activity (18)”; cellular component (CC), including “extracellular membrane-bounded organelle (12),” “outer membrane-bounded periplasmic space (22),” “anchored component of membrane (54),” “microvillus membrane (13),” “periplasmic space (28),” “apical part of cell (41),” “division septum (40),” “growing cell tip (23),” “NELF complex (5),” “box H/ACA snoRNP complex (11)”; biological process (BP), including “negative regulation of anion channel activity by blue light (11),” “streptomycin biosynthetic process (15),” “nicotinamide nucleotide metabolic process (14),” “phototropism (11),” “fungal-type cell wall beta-glucan biosynthetic process (17),” “protein transport by the Sec complex (12),” “response to vitamin D (15),” “hyaluronan catabolic process (9),” “protein secretion by the type II secretion system (14),” “transpiration (15).” KEGG enrichment analysis (Supplementary Tables S6–S8) revealed that the inactivation of *Icwc-1* significantly affected the expression of many genes enriched in the “amino sugar and nucleotide sugar metabolism (19),” “valine, leucine and isoleucine degradation (12),” “nitrogen metabolism (8),” “ABC transporters (10),” “purine metabolism (17),” “cyanoamino acid metabolism (7),” “propanoate metabolism (8),” “methane metabolism (8),” “tyrosine metabolism (11),” “pentose and glucuronate interconversions (6),” “thiamine metabolism (6),” “other glycan degradation (4),” “glycine, serine and threonine metabolism (13),” “ubiquitin mediated proteolysis (13),” “porphyrin and chlorophyll metabolism (6),” “nicotinate and nicotinamide metabolism (7),” “beta-Alanine metabolism (7),” “butanoate metabolism (6),” “ubiquinone and other terpenoid-quinone biosynthesis (4),” “arginine and proline metabolism (9)” (Figures 5B,C). The tendency of gene expression uncovered by transcriptomic analysis was verified by QRT-PCR detection (Supplementary Figure S2).

## 4. Discussion

Fruiting body development is a crucial phase of the mushroom life cycle and depends on various phenotypic and genotypic traits. Fruiting body formation is an important manifestation of the production value of mushrooms, and the formation of fruiting bodies mainly depends on environmental and genotypic factors. Light is one of the most prominent abiotic factors affecting the overall mushroom growth and development, especially in fruiting body development and pigment formation. Previous studies have focused on the effects of light quality on different stages of fungal development (Liu et al., 2019), but few studies have been undertaken at the genetic level.

Light response in model fungi, *Neurospora crassa* is mediated by the WC-1 and WC-2 proteins acting in a complex called the White-Collar-Complex (WCC). The WC-1 protein utilizes FAD (flavin-adenine dinucleotide) as a chromophore. A chromophore is the light-absorbing component in photoreceptor complex. The LOV (light-oxygen-voltage) domain of WC-1 covalently binds FAD at an active cysteine residue upon light exposure. WC-1 contains a Zn finger domain (GATA-like transcription factor domain), two PAS domains that modulate protein interactions, and a putative transcriptional activation domain. The WC-1 homolog can participate not only in blue light but also red light in *Aspergillus nidulans* (Dasgupta et al., 2016). In this study, we cloned the *Icwc-1* gene, the homolog of the blue-light photoreceptor of *N. crassa* and attempted to investigate its effect on the growth and morphological development of mushroom *I. cicadae* (Ponting and Aravind, 1997; Yang and Dong, 2014). The results indicated that the *Icwc-1* gene was highly conserved in different fungal species. In this study, we first used genetic methods to obtain mutants of the blue light gene *Icwc-1* of *I. cicadae* and carried out related biological experiments. The decreased number of conidia after the knockout of the *Icwc-1* gene indicates that the *Icwc-1* gene could promote the production of conidia during the development and asexual reproduction stage of *I. cicadae*. Higher production of conidia, might help in the hyphae kink to form fruiting bodies. It was observed that the aerial mycelium of the mutant strain ∆*Icwc-1* was denser but less likely to form pin-head structural primordium of synnemata. Moreover, the complement strain ∆*Icwc-1*-C showed recovered phenotypes in fruiting body formation and mycelia growth. Our results are consistent with the study conducted on fruit body development in *S. commune* by Ohm et al. (2013). The authors proposed that *Scwc-1* plays an important role in mycelial aggregation and fruiting body maturation. In the mechanism of fruiting body formation (Ohm et al., 2013), ScWC-1 protein acts as a photoreceptor that can receive light signals to regulate the activity of downstream transcription factors, promoting the synthesis of related proteins during fruiting entity development. The same function of the wc-1 homologous gene has also been found in large fungi such as *C. militaris* and *C. sinensis* (Yang et al., 2013; Yang and Dong, 2014).

The cordycepic carotenoids have various bioactive medicinal properties like anticancer, antioxidants and immunomodulatory etc. and also utilized in food industry. In *C. militaris,* content of carotenoid is considered as the parameter of quality standard (Tang et al., 2019; Lou et al., 2020). Previously, it was observed that the carotenoid biosynthesis is affected by the light and imparts the color fungal mycelium and fruiting bodies. Our experimental results showed that the mycelium color of *Icwc-1* mutant strains is significantly different from that of wild-type strains, which is speculated to be caused by the blockade of carotenoid synthesis pathway. Further, we measured the expression of transcription levels of the carotenoid synthesis genes through RT-PCR. The results showed that the expression of *Car1* and *Car2* in the mutant strain was significantly reduced, while the expression of *Car3* and *Car4* did not change significantly. This suggests that in *I. cicadae*, the *Icwc-1* gene may have controlled the amount of carotenoid synthesis by regulating the expression of the *Car1* and *Car2* genes. In another study conducted on *C. militaris,* also showed the similar results. Researchers prepared the *Cmfhp* gene (a light responsible gene) mutant and found that it affects the fruiting bodies and conidia formation along with the reduced production of carotenoids (Lou et al., 2020). The biosynthesis pathway of carotenoids in this important fungus needs to be deeply analyzed in the future.

During the development of macrofungi, there are many genes that regulate the development of fruiting body, and their expression levels can be used to as an important reference for mushroom development. The KEGG enrichment analysis conducted in our study revealed that the inactivation of *Icwc-1* significantly affected regulatory genes participating in various biosynthetic pathways of *I. cicadae* like amino sugar and nucleotide sugar metabolism, ubiquitin mediated proteolysis, ubiquinone and other terpenoid-quinone biosynthesis, arginine and proline metabolism etc., (Supplementary Tables S3, S4). These are the crucial metabolites of fungal growth and development. Previous research showed that several signal pathways and transcription factors participate in fungal light reaction (Dasgupta et al., 2016; Yang et al., 2016). Transcriptome analysis in the present study showed that genes related to MAPK signal pathway and two-component system signal pathway were downregulated seriously (Table 1). In our transcriptome data 4 genes related to two-component system signal pathway, including UM03949 (encoding Transcription initiation factor TFIID subunit) and UM08613 (encoding RPN3, Proteasome regulatory particle subunit) were found to be down-regulated. Two-component signal transduction (TCST) pathways is considered upstream of MAP kinase cascades, play crucial roles in hyphal growth and asexual development in filamentous fungi (Furukawa et al., 2005; Yu et al., 2016; Mohanan et al., 2017). Moreover, 6 genes involved with MAPK signal pathway were down-regulated in ∆*Icwc-1* mutant, including UM03948 [encoding Plasma membrane osmosensor that activates the high osmolarity glycerol (HOG) MAPK signaling pathway] and UM06752 (encoding MAPK component in response to HOG pathway). Literature illustrated that their orthologs have the roles to regulate the secondary metabolism and fruiting body formation (hyphal growth and conidiation) in fungi, such as *Magnaporthe oryzae* (Mehrabi et al., 2008; Liu et al., 2011), *Aspergillus fumigatus* (Rocha et al., 2020), *Trichoderma brevicrassum* (Zhang et al., 2022) and *Neurospora crassa* (Park et al., 2008). Expression of some transcription factors was down-regulated in ∆*Icwc-1* mutant, such as UM07148 (encoding GAL4-like Zn2Cys6 type) and UM01575 (encoding basic leucine zipper (bZIP) family). Homologs of these 2 type of transcription factors are involved in important biological process during growth and fruiting body formation in fungi, including conidium maturation in *Beauveria bassiana* (Chen et al., 2022), conidiation of *Neurospora crassa* (Sun et al., 2012), sexual development and stress responses in *Aspergillus nidulans* (Yin et al., 2013), asexual development in *Aspergillus nidulans* (Etxebeste et al., 2008), differentiation processes and phytotoxin production in *Botrytis cinerea* (Temme et al., 2012), carotenoid synthesis and fruit body formation in *Cordyceps militaris* (Yang et al., 2016). Homolog of UM01321 (encoding Vivid protein) is the component of the transcription factor complex that initiates light-regulated transcriptional responses in *Neurospora* (Chen et al., 2010), and regulates fruiting body formation in *Cordyceps militaris* (Yang et al., 2016). UM02902 encodes guanine nucleotide exchange factors (RhoGEF), RhoGEFs can activate small GTPases of the Rho family (Fort and Blangy, 2017). The function of RhoGTPases is to activate a set of downstream effectors to control cell morphology in eukaryotes. RhoGEF in yeast regulates pheromone response pathway (Hoffman et al., 2000), but rare investigated in filamentous fungi. Due to the fact that the expression of UM02902 was sharply reduced in ∆*Icwc-1* mutant by transcriptome analysis, we infer that it is the putative gene which is related to fruiting body formation. Some putative genes related to the fruiting body formation based on transcriptome analysis are listed in Table 1 and need to be investigated in the future. The results of the present study are consistence with the previous studies showing that the light may stimulate multiple signal pathways leading to the expression of specific transcription factors, which regulate the fungal growth, fruiting body formation and secondary metabolites synthesis. Considering that the synnemata is the main part used for human consumption in this edible fungus, but the molecular mechanisms of synnematal formation is still unclear. Our study provides some candidate gene which need to be assessed in the future for better understanding of the molecular mechanisms of synnematal formation. This may also be useful for molecular breeding improvement on this important fungus.

**Table 1:** some genes putatively related to fruiting body formation based on transcriptome analysis in *Isaria cicadae.*

Protein	log2FC	Protein Length	eggNOG description	Biological process
UM09567	−2.56	236	BTB POZ domain protein	transcriptional regulator
UM01321	−12.76	206	Vivid PAS protein	Response to light
UM07104	−6.40	153	HD domain	cyanamide hydratase in yeast
UM02902	−4.92	643	RhoGEF domain	Activation of GTPases
UM08153	−1.65	1,175	Histidine kinase	Two-component system
UM01553	−1.47	469	Cytochrome c oxidase assembly protein	Two-component system
UM01404	−1.42	480	Glutamine synthetase	Two-component system
UM04757	−1.25	937	Protein tyrosine phosphatase	Two-component system
UM06644	−1.75	553	Translation initiation factor	MAPK signaling pathway
UM02577	−1.25	1949	Belongs to the PI3 PI4-kinase family	Phosphatidylinositol signaling system, related to MAPK
UM06752	−1.22	312	Plasma membrane osmosensor that activates the high osmolarity glycerol (HOG) MAPK signaling pathway	MAPK signaling pathway
UM03948	−1.44	672	Transcription factor	MAPK signaling pathway
UM04982	−1.29	735	Spa2 homology domain (SHD) of GIT	MAPK signaling pathway
UM01575	−1.11	564	(bZIP) family	transcription factor
UM07148	−1.80	918	GAL4-like Zn2Cys6 type	transcription factor
UM03949	−1.32	1,169	Transcription initiation factor	Related to two-component system signal pathway

Here, in this study, we identified a blue light receptor gene *Icwc-1* in Ascomycetes fungi, *I. cicadae* as a novel regulator of synnematal development by providing the following lines of evidence: (1) the transcription of *Icwc-1* was highly induced during the development of fruiting body; (2) some genes, related to fruiting body development, were positively regulated by *Icwc-1*, such as anchored component of membrane (54 related genes), apical part of cell (41 related genes) and division septum (40 related genes). Taken together, our results demonstrated that the *Icwc-1* gene plays a critical role in the growth and development in *I. cicadae*, especially during the formation of fruiting bodies and carotenoid biosynthesis. When the *Icwc-1* gene is knocked out, it results in the inability of *I. cicadae* to form synnemata and conidia formation, which is consistent with the previous studies in *C. cinerea* (Terashima et al., 2005), *S. commune* (Ohm et al., 2013) and *C. militaris* (Yang and Dong, 2014; Lou et al., 2020). In future work, exploring the specific mechanism and factors responsible for the fruiting body development, especially the role of other light responsive genes along with *Icwc-1* on fruiting body development, is an important direction.

## Data availability statement

The original contributions presented in the study are publicly available. This data can be found at: GenBank, OP675621.

## Author contributions

LS: conceptualization, methodology, data curation, investigation, writing — original draft, reviewing and editing. NS: methodology, data curation, writing- reviewing and editing. YG: methodology, data curation, investigation, writing — reviewing and editing. DL: methodology, data curation, writing — reviewing and editing. WC: supervision, resources, funding acquisition. YS: methodology, writing — review and editing. FL: supervision, writing — review and editing. JL: conceptualization, supervision, writing — review and editing. HW: conceptualization, supervision, writing — review and editing, funding acquisition. All authors contributed to the article and approved the submitted version.

## Funding

This work is supported by a grant Organism Interaction from Zhejiang Xianghu Laboratory to FCL. This research was supported by Zhejiang Science and Technology Major Program on Agricultural New Variety Breeding (grant no. 2021C02073-9).

## Conflict of interest

The authors declare that the research was conducted in the absence of any commercial or financial relationships that could be construed as a potential conflict of interest.

## Publisher’s note

All claims expressed in this article are solely those of the authors and do not necessarily represent those of their affiliated organizations, or those of the publisher, the editors and the reviewers. Any product that may be evaluated in this article, or claim that may be made by its manufacturer, is not guaranteed or endorsed by the publisher.

## Footnotes

^1^ http://law.foodmate.net/show-206341.html

^2^ http://www.ncbi.nlm.nih.gov/BLAST/

## References

[ref1] Bolger A. M. Lohse M. Bjoern U. (2014). Trimmomatic: a flexible trimmer for Illumina sequence data. Bioinformatics 30, 2114–2120. doi: 10.1093/bioinformatics/btu170, PMID: 24695404 PMC4103590

[ref2] Cantalapiedra C. P. Hernándezplaza A. Letunic I. Bork P. Jaime H. (2021). eggNOG-mapper v2: functional annotation, orthology assignments, and domain prediction at the metagenomic scale. Mol. Biol. Evol. 38, 5825–5829. doi: 10.1093/molbev/msab293, PMID: 34597405 PMC8662613

[ref3] Chen C.-H. DeMay B. Gladfelter A. S. Dunlapa J. C. Loros J. J. (2010). Physical interaction between VIVID and white collar complex regulates photoadaptation in *Neurospora*. PNAS 107, 16715–16720. doi: 10.1073/pnas.1011190107/-/DCSupplemental, PMID: 20733070 PMC2944764

[ref4] Chen J.-F. Tan J.-J. Wang J.-Y. Mao A.-J. Xu X.-P. Zhang Y.. (2022). The zinc finger transcription factor BbCmr1 regulates Conidium maturation in *Beauveria bassiana*. Microbiol. Spectr. 10:e02066-21. doi: 10.1128/spectrum.02066-21, PMID: 35138172 PMC8826823

[ref5] Dasgupta A. Fuller K. K. Dunlap J. C. Loros J. J. (2016). Seeing the world differently: variability in the photosensory mechanisms of two model fungi. Environ. Microbiol. 18, 5–20. doi: 10.1111/1462-2920.13055, PMID: 26373782 PMC4757429

[ref6] Davidson N. M. Alicia O. (2014). Corset: enabling differential gene expression analysis for de novo assembled transcriptomes. Genome Biol. 15:410. doi: 10.1186/s13059-014-0410-6, PMID: 25063469 PMC4165373

[ref7] Doyle J. J. Doyle J. L. (1987). A rapid DNA isolation procedure for small quantities of fresh leaf tissue. Phytochem Bull. 19, 11–13.

[ref8] Estrada A. F. Maier D. Scherzinger D. Avalos J. Al-Babili S. (2008). Novel apocarotenoid intermediates in *Neurospora crassa* mutants imply a new biosynthetic reaction sequence leading to neurosporaxanthin formation. Fungal Genet. Biol. 45, 1497–1505. doi: 10.1016/j.fgb.2008.09.001, PMID: 18812228

[ref9] Etxebeste O. Ni M. Garzia A. Kwon N. J. Fischer R. Yu J. H.. (2008). Basic-zipper-type transcription factor FlbB controls asexual development in *Aspergillus nidulans*. Eukaryot. Cell 7, 38–48. doi: 10.1128/EC.00207-07, PMID: 17993569 PMC2224158

[ref10] Fort P. Blangy A. (2017). The evolutionary landscape of Dbl-like RhoGEF families: adapting eukaryotic cells to environmental signals. Genome Biol. Evol. 9, 1471–1486. doi: 10.1093/gbe/evx100, PMID: 28541439 PMC5499878

[ref11] Fujita T. Inoue K. Yamamoto S. Ikumoto T. Sasaki S. Toyama R.. (2010). Fungal metabolites. Part 11. A potent immunosuppressive activity found in *Isaria sinclairii* metabolite. J. Antibiot. (Tokyo) 47, 208–215. doi: 10.7164/antibiotics.47.208, PMID: 8150717

[ref12] Furukawa K. Hoshi Y. Maeda T. Nakajima T. Abe K. (2005). *Aspergillus nidulans* HOG pathway is activated only by two-component signalling pathway in response to osmotic stress. Mol. Microbiol. 56, 1246–1261. doi: 10.1111/j.1365-2958.2005.04605.x, PMID: 15882418

[ref13] Grabherr M. G. Haas B. J. Yassour M. Levin J. Z. Thompson D. A. Amit I.. (2011). Full-length transcriptome assembly from RNA-Seq data without a reference genome. Nat. Biotechnol. 29, 644–652. doi: 10.1038/nbt.1883, PMID: 21572440 PMC3571712

[ref04] Guo M. Guo S. Yang H. Ning B. Dong C. (2016). Comparison of Major Bioactive Compounds of the Caterpillar Medicinal Mushroom, Cordyceps militaris (Ascomycetes), Fruiting Bodies Cultured on Wheat Substrate and Pupae. Int. J. Med. Mushrooms 18, 327–336. doi: 10.1615/IntJMedMushrooms.v18.i4.60 27481299

[ref14] Hoffman G. A. Garrison T. R. Dohlman H. G. (2000). Endoproteolytic processing of Sst2, a multidomain regulator of G protein signaling in yeast. J. Biol. Chem. 275, 37533–37541. doi: 10.1074/jbc.M005751200, PMID: 10982801

[ref15] Huang Y. S. Wang X. Feng Z. Cui H. Liu Y. N. (2020). *Cordyceps cicadae* prevents renal tubular epithelial cell apoptosis by regulating the SIRT1/p53 pathway in hypertensive renal injury. Evid. Based Complement. Alternat. Med. 2020, 1–13. doi: 10.1155/2020/7202519, PMID: 32419819 PMC7201718

[ref16] Jiao X. Zhi-Cheng T. Zhong-Yuan S. Xing-Jia S. Shun-Ming T. (2021). *Cordyceps cicadae* polysaccharides inhibit human cervical cancer hela cells proliferation via apoptosis and cell cycle arrest. Food Chem. Toxicol. 148:111971. doi: 10.1016/j.fct.2021.111971, PMID: 33421460

[ref17] Jike L. U. Guofeng G. U. Hao L. Jin Z. Wang X. U. (2016). Characterization and *in vitro* antioxidant activity of a polysaccharide from *Cordyceps sobolifera*. J. Food Process. Preserv. 40, 447–452. doi: 10.1111/jfpp.12622

[ref18] Khang C. H. Park S. Y. Rho H. S. Lee Y. H. Kang S. (2006). Filamentous fungi (*Magnaporthe grisea* and *Fusarium oxysporum*). Methods Mol. Biol. 344, 403–420. doi: 10.1385/1-59745-131-2:403, PMID: 17033082

[ref19] Kumar S. Stecher G. Koichiro T. (2016). MEGA7: molecular evolutionary genetics analysis version 7.0 for bigger datasets. Mol. Biol. Evol. 33, 1870–1874. doi: 10.1093/molbev/msw054, PMID: 27004904 PMC8210823

[ref20] Kuo Y. C. Wen S. C. Chou C. J. Tsai W. J. (2003). Activation and proliferation signals in primary human T lymphocytes inhibited by ergosterol peroxide isolated from *Cordyceps cicadae*. Br. J. Pharmacol. Chemother. 140, 895–906. doi: 10.1038/sj.bjp.0705500, PMID: 14504132 PMC1574094

[ref21] Langmead B. Salzberg S. (2011). Fast gapped-read alignment with Bowtie 2. Nat. Methods 9, 357–359. doi: 10.1038/nmeth.1923, PMID: 22388286 PMC3322381

[ref22] Latini S. Pedata F. (2010). Adenosine in the central nervous system: release mechanisms and extracellular concentrations. J. Neurochem. 79, 463–484. doi: 10.1046/j.1471-4159 11701750

[ref23] Li L. Zhang T. Li C. Lu X. Li N. Hou T. L.. (2019). Potential therapeutic effects of *Cordyceps cicadae* and *Paecilomyces cicadae* on adenine-induced chronic renal failure in rats and their phytochemical analysis. Drug Des. Devel. Ther. 13, 103–117. doi: 10.2147/DDDT.S180543, PMID: 30587931 PMC6304081

[ref01] Linden H. Ballario P. Macino G. (1997). Blue light regulation in Neurospora crassa. Fungal Genet. Biol. 22, 141–150. doi: 10.1006/fgbi.1997.1013 9454641

[ref02] Liu Y. He Q. Cheng P. (2003). Photoreception in Neurospora: a tale of two White Collar proteins. Cellular and Molecular Life Sciences 60, 2131–2138. doi: 10.1007/s00018-003-3109-5 14618260 PMC11146069

[ref03] Liu S. Q. (2008). Determination of the Active Ingredient Produced in the Artificial Cultivated Cordyceps sobolifera. Journal of Anhui Agricultural Sciences 36, 429–419.

[ref24] Liu K. Wang F. Liu G. Dong C. (2019). Effect of environmental conditions on Synnema formation and nucleoside production in cicada flower, *Isaria cicadae* (ascomycetes). Int. J. Med. Mushrooms 21, 59–69. doi: 10.1615/IntJMedMushrooms.2018029506, PMID: 30806256

[ref25] Liu W. Zhou X. Li G. Li L. Kong L. Wang C.. (2011). Multiple plant surface signals are sensed by different mechanisms in the Rice blast fungus for appressorium formation. PLoS Pathog. 7:e1001261. doi: 10.1371/journal.ppat.1001261, PMID: 21283781 PMC3024261

[ref26] Liu W. S. Zhu X. H. Lei M. G. Xia Q. Y. Botella J. R. Zhu J. K.. (2015). A detailed procedure for CRISPR/Cas9-mediated gene editing in *Arabidopsis thaliana*. Sci. Bull. 60, 1332–1347. doi: 10.1007/s11434-015-0848-2

[ref27] Lou H. W. Zhao Y. Chen B. X. Yu Y. H. Tang H. B. Ye Z. W.. (2020). Cmfhp gene mediates fruiting body development and carotenoid production in *Cordyceps militaris*. Biomol. Ther. 10:410. doi: 10.3390/biom10030410, PMID: 32155914 PMC7175373

[ref28] Lou H.-W. Zhao Y. Tang H.-B. Ye Z.-W. Wei T. Lin J.-F.. (2019). Transcriptome analysis of *Cordyceps militaris* reveals genes associated with carotenoid synthesis and identification of the function of the Cmtns gene. Front. Microbiol. 10:2105. doi: 10.3389/fmicb.2019.02105, PMID: 31552008 PMC6746990

[ref29] Lu J. P. Cao H. J. Zhang L. L. Huang P. Y. Lin F. C. (2014). Systematic analysis of Zn2Cys6 transcription factors required for development and pathogenicity by high-throughput gene knockout in the rice blast fungus. PLoS Pathog. 10:e1004432. doi: 10.1371/journal.ppat.1004432, PMID: 25299517 PMC4192604

[ref30] Lu M. Y. Chen C. C. Lee L. Y. Lin T. W. Kuo C. F. (2015). N(6)-(2-hydroxyethyl)adenosine in the medicinal mushroom *Cordyceps cicadae* attenuates lipopolysaccharide-stimulated pro-inflammatory responses by suppressing TLR4-mediated NF-κB signaling pathways. J. Nat. Prod. 78, 2452–2460. doi: 10.1021/acs.jnatprod.5b00573, PMID: 26394068

[ref31] Mehrabi R. Ding S. Xu J.-R. (2008). MADS-box transcription factor Mig1 is required for infectious growth in *Magnaporthe grisea*. Eukaryot. Cell 7, 791–799. doi: 10.1128/EC.00009-08, PMID: 18344407 PMC2394974

[ref32] Mohanan V. C. Chandarana P. M. Chattoo B. B. Patkar R. N. Manjrekar J. (2017). Fungal histidine phosphotransferase plays a crucial role in Photomorphogenesis and pathogenesis in *Magnaporthe oryzae*. Front. Chem. 5:31. doi: 10.3389/fchem.2017.00031, PMID: 28580356 PMC5437211

[ref33] Ohm R. A. Aerts D. Wsten H. A. B. Lugones L. G. (2013). The blue light receptor complex WC-1/2 of *Schizophyllum commune* is involved in mushroom formation and protection against phototoxicity. Environ. Microbiol. 15, 943–955. doi: 10.1111/j.1462-2920, PMID: 22998561

[ref34] Olatunji O. J. Feng Y. Olatunji O. O. Tang J. Ouyang Z. Su Z. (2016a). Cordycepin protects PC12 cells against 6-hydroxydopamine induced neurotoxicity via its antioxidant properties. Biomed. Pharmacother. 81, 7–14. doi: 10.1016/j.biopha.2016.03.009, PMID: 27261571

[ref35] Olatunji O. J. Yan F. Olatunji O. Jian T. Zhen O. Su Z.. (2016b). Neuroprotective effects of adenosine isolated from *Cordyceps cicadae* against oxidative and ER stress damages induced by glutamate in PC12 cells. Environ. Toxicol. Pharmacol. 44, 53–61. doi: 10.1016/j.etap.2016.02.009, PMID: 27114365

[ref36] Park G. Pan S. Q. Borkovich K. A. (2008). Mitogen-activated protein kinase cascade required for regulation of development and secondary metabolism in *Neurospora crassa*. Eukaryot. Cell 7, 2113–2122. doi: 10.1128/EC.00466-07, PMID: 18849472 PMC2593188

[ref37] Ponting C. P. Aravind L. (1997). PAS: a multifunctional domain family comes to light. Curr. Biol. 7, R674–R677. doi: 10.1016/s0960-9822(06)00352-6, PMID: 9382818

[ref38] Qu Y. M. Cao H. J. Huang P. Y. Wang J. Liu X. H. Lu J. P.. (2022). A kelch domain cell end protein, PoTea1, mediates cell polarization during appressorium morphogenesis in *Pyricularia oryzae*. Microbiol. Res. 259:126999. doi: 10.1016/j.micres.2022.126999, PMID: 35305442

[ref39] Rocha M. C. Fabri J. H. T. M. Simões I. T. Silva-Rocha R. Hagiwara D. da Cunha A. F.. (2020). The cell wall integrity pathway contributes to the early stages of *Aspergillus fumigatus* asexual development. Appl. Environ. Microbiol. 86:e02347-19. doi: 10.1128/AEM.02347-19, PMID: 32005734 PMC7082570

[ref40] Smiderle F. R. Baggio C. H. Borato D. G. Santana-Filho A. P. Sassaki G. L. Marcello I.. (2014). Anti-inflammatory properties of the medicinal mushroom *Cordyceps militaris* might be related to its linear (1→3)-β-D-glucan. PLoS One 9:e110266. doi: 10.1371/journal.pone.0110266, PMID: 25330371 PMC4201515

[ref41] Sun Y. Wink M. Pan W. Lu H. Liang Z. (2017). Biological characteristics, bioactive components and antineoplastic properties of sporoderm-broken spores from wild *Cordyceps cicadae*. Phytomedicine 36, 217–228. doi: 10.1016/j.phymed.2017.10.004, PMID: 29157818

[ref42] Sun X. Yu L. Lan N. Wei S. Yu Y. Zhang H.. (2012). Analysis of the role of transcription factor VAD-5 in conidiation of *Neurospora crassa*. Fungal Genet. Biol. 49, 379–387. doi: 10.1016/j.phymed.2017.10.004, PMID: 22445960

[ref43] Tang H. Ye Z. W. Liu C. Guo L. Q. Lin J. F. Wang H.. (2019). Increasing of the contain of carotenoids in Caterpillar mushroom, *Cordyceps militaris* (ascomycetes) by using the fungal elicitors cultivation. Int. J. Med. Mushrooms 21, 1181–1191. doi: 10.1615/IntJMedMushrooms.2019032998, PMID: 32464011

[ref44] Temme N. Oeser B. Massaroli M. Heller J. Simon A. Collado I. G.. (2012). BcAtf1, a global regulator, controls various differentiation processes and phytotoxin production in *Botrytis cinerea*. Mol. Plant Pathol. 13, 704–718. doi: 10.1111/J.1364-3703.2011.00778.X, PMID: 22293085 PMC6638710

[ref45] Terashima K. Yuki K. Muraguchi H. Akiyama M. Kamada T. (2005). The *dst1* gene involved in mushroom photomorphogenesis of *Coprinus cinereus* encodes a putative photoreceptor for blue light. Genetics 171, 101–108. doi: 10.1534/genetics.104.040048, PMID: 15956671 PMC1456503

[ref05] Wu T. Hu C. Xie B. Zhang L. Li S. (2019). A Single Transcription Factor (PDD1) Determines Development and Yield of Winter Mushroom (Flammulina velutipes). Applied and Environmental Microbiology 85:e01735-19. doi: 10.1128/aem.01735-19 31604770 PMC6881794

[ref46] Wang Y. B. He P. F. He L. Huang Q. R. Cheng J. W. Li W. Q.. (2019). Structural elucidation, antioxidant and immunomodulatory activities of a novel heteropolysaccharide from cultured *Paecilomyces cicadae* (Miquel.) Samson. Carbohydr. Polym. 216, 270–281. doi: 10.1016/j.carbpol.2019.03.104, PMID: 31047067

[ref47] Yang T. Dong C. (2014). Photo morphogenesis and photo response of the blue-light receptor gene Cmwc-1 in different strains of *Cordyceps militaris*. FEMS Microbiol. Lett. 352, 190–197. doi: 10.1111/1574-6968.12393, PMID: 24484244

[ref48] Yang T. Guo M. Yang H. Guo S. Dong C. (2016). The blue-light receptor CmWC-1 mediates fruit body development and secondary metabolism in *Cordyceps militaris*. Appl Microbiol. Biotechnol. 100, 743–755. doi: 10.1007/s00253-015-7047-6, PMID: 26476643

[ref49] Yang T. Xiong W. Dong C. (2013). Cloning and analysis of the *Oswc-1* gene encoding a putative blue light photoreceptor from *Ophiocordyceps sinensis*. Mycoence 55, 241–245. doi: 10.1016/j.myc.2013.09.003

[ref50] Yin W.-B. Reinke A. W. Tamas E. M. Chiang Y.-M. Keating A. E. Pocsi I.. (2013). bZIP transcription factors affecting secondary metabolism, sexual development and stress responses in *Aspergillus nidulans*. Microbiology 159, 77–88. doi: 10.1099/mic.0.063370-0, PMID: 23154967 PMC3542729

[ref51] Yu Z. Armant O. Fischer R. (2016). Fungi use the SakA (HogA) pathway for phytochrome-dependent light signaling. Nat. Microbiol. 1:16019. doi: 10.1038/NMICROBIOL.2016.19, PMID: 27572639

[ref52] Yu G. C. Wang L. G. Han Y. Y. He Q. Y. (2012). clusterProfiler: an R package for comparing biological themes among gene clusters. OMICS J. Integr. Biol. 16, 284–287. doi: 10.1089/omi.2011.0118, PMID: 22455463 PMC3339379

[ref53] Yu J. W. Xu H. J. Mo Z. H. Zhu H. L. Mao X. B. (2009). Determination of myriocin in natural and cultured *Cordyceps cicadae* using 9-fluorenylmethyl chloroformate derivatization and high-performance liquid chromatography with UV-detection. Anal. Sci. 25, 855–859. doi: 10.2116/analsci.25.855, PMID: 19609022

[ref06] Zeng W.-B. Yu H. Ge F. Yang J.-Y. Chen Z.-H. Wang Y.-B.. (2014). Distribution of nucleosides in populations of Cordyceps cicadae. Molecules 19, 6123–6141. doi: 10.3390/molecules19056123 24830714 PMC6271799

[ref54] Zhang X. Hu Q. Weng Q. (2019). Secondary metabolites (SMs) of *Isaria cicadae* and *Isaria tenuipes*. RSC Adv. 9, 172–184. doi: 10.1039/c8ra09039d, PMID: 35521576 PMC9059538

[ref07] Zhang Y. Zhuang W.-Y. (2022). MAPK Cascades Mediating Biocontrol Activity of Trichoderma brevicrassum Strain TC967. Journal of agricultural and food chemistry 70, 2762–2775. doi: 10.1016/j.jes.2022.02.040 35191703

