# Supplementary material for: Role of the blue light receptor gene Icwc-1 in mycelium growth and fruiting body formation of Isaria cicadae
Source: Front Microbiol. 2023 Jan 10;13:1038034. doi: 10.3389/fmicb.2022.1038034 (PMC9871644; doi:10.3389/fmicb.2022.1038034)
Supplement: Supplementary file 1 [file Data_Sheet_1.docx]

Supplementary Material

# Supplementary Data

**1.1 Nucleotides sequence of *Icwc-1* (Opening reading frame and introns).** The coding sequence of *Icwc-1* is 2940 bp, which containing two exons (in black) interrupted by a 62 bp intron (in blue)

ATGGAAGGATACTATCCTTCCAATCAGTTGCCTACTCAGGAACAGCAGAGGCAGCGACAGAACAATATCAGCAACACgacacagcagcagcagcagcagcaacaacaacaacaaatgCCTCCTAGTATGAGTATGCCGCACGGCAACACATTTACGAGTCAAATGATGGGCAATCCAACACTACTACAGCGCCGTGCGAGCGCCTCCCTAGGCTTCGGGGCGCGGCAACTGTCGGACGAGGTAGACGCCAGACGCATGTCATTGGCTGTCGAGCAAGGAGCCATGAATCCGCCGTCCACTACGGGGCCTGGCAATTTCATGGGGTTTCAGCCTTCAGCGCAACATCTCAACAATTTTTCGATGCTGGATACCTCGAACATGGGCAGCATGATGCCTGATGGCGATGGTTTTCCGAACTTATCTCCCGATGCCATGGGAAACTTGGTATCTGCTCAGTTCGCCAATATTAACATGGGAGCAATGTTTCCCAGTAGTTCAAGTGCCATACTTGGCACACAAACGTCACCAACCGTCATTCAGTTATCCATGCCCTCCAACGATACTATTCACTTTAGCAATACGGCAGAATATGGCGCCTTTACCAACCCGCGTGACAATACGGTCCCGGCTCCCCTTCAAATGAGCCAGCTTGAAACGCCCACGAATCCATCTCATGATCCAAGGCTTGGCGCGACTGCTGTTGGTGTTGATCAATCCATATCATCAATCCCCCATCATGAATCATCAGCTTGCTCCGCCGCTTCCCCTGTGCAAGAAAAATCGTCATCAACCGGACCTACCCAACCAAGCACCTCATCTGTCGCTACGACCCCAAACGCTCCAGCGACTTCCAGGGATCTTCGCGAAAGATCGATTTACTCCAAAAGCGGCTTTGATATGCTCAAAGCTCTCTGGCTTGTTGCATCCAGAAAGAATCCTTCGATTGATCTAGGGGCAGTAGACATGTCGTGTGCTTTTGTTGTGTGCGACATCACGATGAACGATTGCCCTATCATATATGTCTCTGATAATTTTCAAAACTTGACTGGCTACAGCAGCCATGATATTGTTGGCCAAAATTGCCGTTTTCTTCAAGCACCGGACGGGAAAGTTGAGGCTGGAACTAAGCGCGAATTCGTTGATAACGGCGCAGTTTTCAACTTGAAGAAGATGATACAAGAGAGACGGGAGGTGCAACAAAGTCTCATTAACTACCGAAAAGGCGGCAAGCCGTTTCTTAACCTTCTGACAATGATTCCGATACCCTGGGAAACGGATGAGATCCGCTATTTCATTGGATTCCAGATCGACCTTGTCGAATGCCCCGACGCTATTGCCGGTTCTGAATTTGGTGGCGTCGCCGTGAATTATAAGCACAGCGATATCGGCCAGTACATATGGACACCACCTTCCTCCGCTTTCTGGGAAGCCGACAACGGACAGACTTTGGGCGTTGATGATGTGTCGACAATTTTGCAACAGTTCAATGCCAAAGGTATCGCGTCCGACTGGCATCGTCAGTCTTGGGATAAGATGCTGCTTGAGAATTGCGACGACGTGATCCATGTACTCTCTCTCAAGGGCCTCTTCCTCTATCTCTCGCCAGCCTGCAAACGCGTCTTGGAGTATGATGCCGCGGAGTTGGTTGGCAACTCATTATCATCAATCTGCCATCCTTCTGACATTGTCCCTGTTACACGCGAATTGAAAGATGCGACCACCGGCGATCAAGTAAATATTGTTTTCAGGATTCGACGAAAGCAAAGTGGATACACGTGGTTTGAGAGCCATGGTTCTCTGTACATTGAGCAAGGTAAAGGCCGCAAATGCATCATTCTCGTCGGTAGAAAGCGACCTGTGTTCTCCATCAGTCGCCGAAATATTGAATCCAATGGTGGCATCGGTGATAGCGAGTTGTGGACCAAATTATCTACCTCCGGCACCTTCCTCTACGTATCGTCGAACGTGCGATCTCTTCTTGACTTACAGCCCGAATCCCTGGTCGCCACAAGCATTCAGGAACTGATGCGGAAAGATTCGAGGCCGGAGTTCGGTCGCACGTTGGAAAAGGCGCGACGCGGAAAGATTGTGACCTGCAAACACGAAGTTCTGAACCGGCGTGGCCAAGGGCTCCAAGCTCAAACTACACTCTATCCCGGAGATGCTACTGAGGGGCAAAAGCCATCTTTCTTGCTTGCTCAGACGAAGCTATTAAAAGCATCCTCAAGAGCGCTTGCGCCAGCTATTTCGACAGCGGGTTCTACGGCTGGTAGATCGGTGCATGGCCAGATTTCCCAgcccggaggcggcggactAGCTCTCGGTAATCAAGACGAGGCACTTGCGTCTGACGACAACATCTTCGATGAGCTGAGGACGACGAAATGTTCGAGCTGGCAATTCGAGCTGCGTCAGATGGAGAAAGTAAACCGAATCCTAGCAGAAGAGCTTGGCGGCTTGCTATCTAgcaagaaaaagaggaagcGGCGCAAGGGAGTCGGCAACGTGGTTCGCGATTGCGCCAATTGCCATACGCGCAATACGCCAGaatggcgtcgaggacccAGTGGCCAGCGCGATCTTTGCAACAGCTGTGGCCTGCGATGGGCCAAACAGGTAAGCCCACCAAAAAGCTCTGTCTGCCCAGCGATGATGAGACTAACGAGCCTCATGTCAGATGGGTCGAGTGTCACCGCGCAACTCTGTACGCAATGACGATTCCCAAAGCCGAAAGTCTGCGTCACCCATACATTCATCACCTCTGCACAAGGAGGTATCTGCAGGCGCAAACTCAACTAATCCTGACGCCAAGAGATCAGCGACAACCGAAACTTCGGCTTCCGCAGATTCCATATCCTCGTctaccaccagccagcccacgAACCGCACTGTCAGTGCTGGGACAGCGATGGCTTCAATTCGAGAAGAGCGAGAAACTAG

**1.2 Predicted protein sequence of IcWC-1.** The predicted protein sequence of IcWC-1 which contains 959 amino acids.

MEGYYPSNQLPTQEQQRQRQNNISNTTQQQQQQQQQQQMPPSMSMPHGNTFTSQMMGNPTLLQRRASASLGFGARQLSDEVDARRMSLAVEQGAMNPPSTTGPGNFMGFQPSAQHLNNFSMLDTSNMGSMMPDGDGFPNLSPDAMGNLVSAQFANINMGAMFPSSSSAILGTQTSPTVIQLSMPSNDTIHFSNTAEYGAFTNPRDNTVPAPLQMSQLETPTNPSHDPRLGATAVGVDQSISSIPHHESSACSAASPVQEKSSSTGPTQPSTSSVATTPNAPATSRDLRERSIYSKSGFDMLKALWLVASRKNPSIDLGAVDMSCAFVVCDITMNDCPIIYVSDNFQNLTGYSSHDIVGQNCRFLQAPDGKVEAGTKREFVDNGAVFNLKKMIQERREVQQSLINYRKGGKPFLNLLTMIPIPWETDEIRYFIGFQIDLVECPDAIAGSEFGGVAVNYKHSDIGQYIWTPPSSAFWEADNGQTLGVDDVSTILQQFNAKGIASDWHRQSWDKMLLENCDDVIHVLSLKGLFLYLSPACKRVLEYDAAELVGNSLSSICHPSDIVPVTRELKDATTGDQVNIVFRIRRKQSGYTWFESHGSLYIEQGKGRKCIILVGRKRPVFSISRRNIESNGGIGDSELWTKLSTSGTFLYVSSNVRSLLDLQPESLVATSIQELMRKDSRPEFGRTLEKARRGKIVTCKHEVLNRRGQGLQAQTTLYPGDATEGQKPSFLLAQTKLLKASSRALAPAISTAGSTAGRSVHGQISQPGGGGLALGNQDEALASDDNIFDELRTTKCSSWQFELRQMEKVNRILAEELGGLLSSKKKRKRRKGVGNVVRDCANCHTRNTPEWRRGPSGQRDLCNSCGLRWAKQMGRVSPRNSVRNDDSQSRNDNRNFGFRRFHILVYHQPAHEPHCQCWDSDGFNSRRARN

# Supplementary Figures


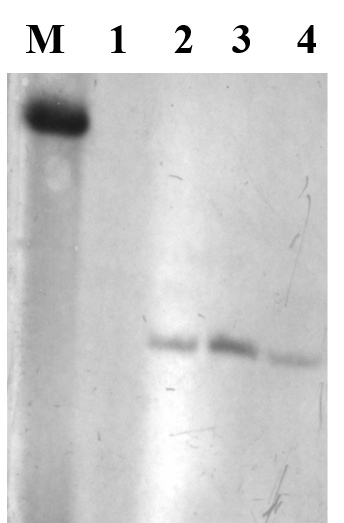


**Figure S1** **Results of Southern blot analysis.**

*Icwc-1* mutants identified via southern blot. Lane 1: WT strain, Lanes 2, 3 and 4: △*Icwc-1* strains. Genomic DNA is digested with *Xho*l for probing HpH in southern blotting hybridization.

**Figure S2 Validation of Transcriptome by q-PCR.**

Expression of 6 down-regulated genes in transcriptome analysis was validated by q-PCR. UM01575: transcription factor; UM03948 and UM06752: component of MAPK cascade; UM00988: ABC transporter; UM03949: Transcription initiation factor TFIID subunit; UM02436: Zinc finger protein.
